# Loss of *ZC4H2*, an Arthrogryposis Multiplex Congenita Associated Gene, Promotes Osteoclastogenesis in Mice

**DOI:** 10.3390/genes15091134

**Published:** 2024-08-28

**Authors:** Liang Zhu, Longlong Zhang, Jingmei Cha, Chaocui Li, Bingyu Mao

**Affiliations:** 1 State Key Laboratory of Genetic Resources and Evolution, Kunming Institute of Zoology, Chinese Academy of Sciences, Kunming 650201, China; zliang689@163.com (L.Z.); 18388004332@163.com (J.C.); licc@mail.kiz.ac.cn (C.L.); 2 Kunming College of Life Science, University of Chinese Academy of Sciences, Kunming 650201, China; 3 Academy of Biomedical Engineering, Kunming Medical University, Kunming 650500, China; zhanglonglong@kmmu.edu.cn

**Keywords:** *ZC4H2*, bone development, osteoclastogenesis, osteoclast differentiation

## Abstract

*ZC4H2* encodes a *C4H2*-type zinc finger protein, mutations of which lead to a spectrum of diseases known as *ZC4H2* associated rare disorders (ZARD). In addition to neurological phenotypes, the most typical symptoms of ZARD are multiple joint contractures of varying degrees, accompanied by abnormal development of muscles and bones, and osteoporosis in some cases. The pathogenic mechanisms of such bone related phenotypes, however, remain unclear. Here, we showed that *ZC4H2* is expressed in the developing bones in mice. *ZC4H2* knockout mice were neonatal-lethal and smaller in size, with reduced calcification of long bones. Upon induced loss of *ZC4H2* postnatally, the femoral bones developed an osteoporosis-like phenotype, with reduced bone mineral density, bone-volume fraction, and trabecular bone number. Knockdown of *ZC4H2* showed no clear effect on the expression of osteogenic differentiation genes in in vitro models using mesenchymal stem cells. Interestingly, *ZC4H2* knockdown significantly enhanced osteoclast differentiation and bone resorption in induced bone marrow-derived macrophages. We further confirmed that the number of osteoclasts in the long bone of *ZC4H2* knockout mice was increased, as well as the expression of the serum bone resorption/osteoporosis marker CTX-1. Our study unveils a new role of *ZC4H2* in osteoclast differentiation and bone development, providing new clues on the pathology of ZARD.

## 1. Introduction

Zinc finger *C4H2*-type containing (*ZC4H2*) is a zinc finger protein, and the *ZC4H2* gene is located on the X chromosome in both mice and humans. In humans, *ZC4H2* has been identified as a causal gene of Wieacker–Wolff syndrome. Affected patients with *ZC4H2* mutations show various clinical phenotypes, including arthrogryposis multiplex congenita (AMC), intellectual disability, and epilepsy, which are now collectively referred to as *ZC4H2*-associated rare disorders (ZARD) [1,2,3,4,5,6]. *ZC4H2* deficiency leads to severe neurodevelopmental impairments in humans and has been shown to be widely involved in neural development in mouse and zebrafish models [2,6,7,8,9,10]. Mechanistically, *ZC4H2* has been shown mostly to work as a stabilizing co-factor of the ubiquitin E3 ligase RNF220 [9,11,12]. RNF220 and *ZC4H2* cooperate to pattern the embryonic ventral neural tube through regulation of Shh/Gli signaling as well as the stability of key transcription factors (Dbx1/2 and Nkx2.2) [11]. They are both required for noradrenergic neuron development in the locus coeruleus (LC), where *ZC4H2* works as an adaptor to bridge RNF220 to its targets, Phox2a/b, for mono-ubiquitination [7]. In addition, *ZC4H2*/RNF220 are also involved in cerebellum development and Shh-group medulloblastoma progression [13].

In addition to the neurological phenotypes, the most typical symptoms of ZARD are multiple joint contractures of varying degrees, accompanied by the abnormal development of muscles and bones, and osteoporosis in some cases [2,4,5,14,15]. Whether *ZC4H2* is directly involved in bone development, however, remains unclear. Bone development and homeostasis are regulated by the balance of osteoblast-mediated bone formation and osteoclast-mediated bone resorption [16,17,18,19]. In this study, *ZC4H2* mutant mice were used to study the roles of *ZC4H2* in bone development. In vivo and in vitro experiments were conducted to investigate the effects of *ZC4H2* deletion on the differentiation and proliferation of osteoblast and osteoclast lineage cells.

## 2. Materials and Methods

### 2.1. Animal Experimentation

All animal procedures conducted in this study were approved by the Institutional Animal Care and Use Committee (IACUC) of Kunming Institute of Zoology, Chinese Academy of Sciences. Mice containing conditional alleles of *ZC4H2* were previously described [11]. They were mated to Vasa-Cre or Rosa26-CreERT2 transgenic mice. Vasa-Cre, which is expressed in the oocyte, would induce complete loss of *ZC4H2* in the *ZC4H2*^fl/Y^ embryos. *ZC4H2*^fl/Y^; Vasa-Cre-positive mice are referred to as *ZC4H2* knockout (KO); *ZC4H2*^+/Y^; Vasa-Cre-positive mice were used as controls (WT). *ZC4H2*^fl/Y^; Rosa26-CreERT2-positive mice induced with tamoxifen are referred to as *ZC4H2* TAM-knockout (TAM-KO); *ZC4H2*^fl/Y^; Rosa26-CreERT2-positive mice without tamoxifen injection were used as controls (WT). For tamoxifen-inducible knockout mice, mice at one-week old were administered an intraperitoneal injection of tamoxifen (75 mg/kg) once daily for five consecutive days. Note that as *ZC4H2* is located on the X chromosome, in this study, male *ZC4H2*^fl/Y^ and female *ZC4H2*^fl/+^; Vasa-Cre mice were used as crossing pairs, and only male *ZC4H2*^fl/Y^; Vasa-Cre mice were used for following phenotypic and cellular analysis, to keep consistent with the *ZC4H2*^+/Y^ littermate controls. The presence of *ZC4H2* floxed and wild-type alleles were detected by PCR using genomic DNA prepared from distal tails. Sequences of the primers used for genotyping are referenced from previously reported [11]. 

### 2.2. Quantitative Real-Time Polymerase Chain Reaction (qRT-PCR)

Total RNA was extracted from cells by Trizol reagent (TianGen, Beijing, China) and was reverse-transcribed into complementary DNA (cDNA) using a PrimeScript RT reagent kit (TaKaRa, Tokyo, Japan) according to the manufacturer’s instructions. qRT-PCR was performed using SYBR Green PCR Master Mix (TaKaRa, Tokyo, Japan) and on the CFX96Touch Real Time PCR system (Bio-Rad, Hercules, CA, USA). Primer sequences used are given in Table 1.

### 2.3. MSCs, BMDM Isolation and In Vitro Differentiation

Mesenchymal stem cells (MSCs) and bone marrow derived macrophages (BMDM) were isolated from 6–8 week-old *ZC4H2*^fl/Y^; Rosa26-CreERT2 mice as previously reported [20]. 4-OH-tamoxifen (4-OHT, 1.5 μM) was used to induce *ZC4H2* knockout in MSCs and BMDM, and ethanol was used as a control. The MSCs and BMDM with or without 4-OHT treatment for 24 h were cultured and induced to osteoblast and osteoclast differentiation, respectively, as previously reported [21,22]. In brief, for in vitro osteoblast differentiation, 2 × 10^5^ MSCs were seeded in 12-well plates and cultured in osteogenic medium with α-MEM medium containing 10% FBS, 1% Penicillin/Streptomycin, 10 mM β-glycerol phosphate, and 50 μg/mL ascorbic acid, and the medium was changed every 2 days. For in vitro osteoclast differentiation, BMDM were seeded in 24-well plates at a density of 1 × 10^5^ cells per well and cultured in osteoclastogenic medium with α-MEM containing 30 ng/mL M-CSF and 100 ng/mL RANKL (PeproTech, Cranbury, NJ, USA); the medium was changed every 2 days.

### 2.4. RNA-Seq Analysis

For RNA-Seq analysis of the differentiated BMDM, cells were collected one week after osteoclast differentiation and RNAs were extracted for transcriptome sequencing. The sequencing library was constructed and sequenced by BGI-Genomics on the Illumina platform. First, the sequencing data were filtered with SOAPnuke (v1.5.2) [23]. Subsequently, the clean reads were mapped to the reference genome GRCm38 (https://www.ncbi.nlm.nih.gov/grc/mouse (accessed on 15 August 2023)) using HISAT2 (v2.0.4) [24]. Next, Bowtie2 (v2.2.5) [25] was used to align the clean reads to the reference coding gene set, followed by gene expression level calculation using RSEM (v1.2.12) [26]. Differential expression analysis was performed using DESeq2 (v1.4.5) [27] with a Q value ≤ 0.05 and an absolute fold change ≥ 1.5. Kyoto Encyclopedia of Genes and Genomes (KEGG, https://www.kegg.jp/ (accessed on 15 August 2023)) enrichment analysis of annotated differentially expressed genes was conducted using Phyper (https://en.wikipedia.org/wiki/Hypergeometric_distribution (accessed on 15 August 2023)) based on the Hypergeometric test. The significance levels of terms and pathways were corrected by Q value using a rigorous threshold (Q value ≤ 0.05) according to Bonferroni [28,29].

### 2.5. Von Kossa Staining

Femur sections were deparaffinized and rehydrated, stained with von Kossa silver solution under strong light for 15–60 min. The sections were then washed, fixed with sodium thiosulfate solution for 2 min, counterstained with hematoxylin and eosin (HE), mounted, and scanned using a digital slide scanning system (Olympus Corporation, Tokyo, Japan). 

### 2.6. Skeletal Staining

Alcian blue and Alizarin red S (ARS) staining of mouse skeletons was performed according to previous reports [21]. The samples were fixed in 95% ethanol for 24 h, stained with Alcian blue staining solution for 12–18 h, and washed 5 times with 95% ethanol. For ARS staining, the samples are treated with 2% KOH for 3–4 h, then stained with ARS staining solution for 3–4 h. Subsequently, they were washed 5 times with 1% KOH/20% glycerol solution. Images are quantitatively analyzed using Nikon software (NIS-Elements D (v5.10.00), Nikon, Tokyo, Japan).

### 2.7. Micro-Computed Tomography (Micro-CT)

Mouse femurs of control and TAM-*ZC4H2* KO groups at 3 months old were collected and scanned using a SkyScan1276 (Bruker, Kartuizersweg, Belgium) micro-CT at 8 μm resolution for quantitative analysis. The turn on voltage was 85 kV and current density was 150 μA. Analyses of bone mineral density (BMD), bone volume-to-total volume ratio (BV/TV), trabecular number (Tb.N), and trabecular separation (Tb.Sp) are conducted in the region 0–100 layers below the growth plate. Analyses of cortical thickness (Ct.Th) and cortical bone mineral density (Ct.BMD) are conducted in the region 300–400 layers below the growth plate. Volumetric reconstructions and analyses were performed using built-in software CTvox (v3.3.0r1383) and CTAn (v1.17.7.2), respectively.

### 2.8. Alkaline Phosphatase (ALP) and Alizarin Red S (ARS) Staining 

For analysis of the osteoblast differentiated MSCs, after 8 days of induction, ALP staining and quantification were performed using an ALP staining kit (Solarbio, Beijing, China) and an ALP quantification kit (Beyotime, Shanghai, China), respectively. After about three weeks of induction, ARS staining was performed using an ARS staining kit (Solarbio, Beijing, China) and quantified using 10% cetylpyridinium chloride (CPC). The quantified ALP and ARS activities were normalized to protein content. 

### 2.9. Tartrate Resistant Acid Phosphatase (TRAP) Staining Assay

For analysis of the osteoclast differentiated BMDM, after 7 days of induction, TRAP staining was performed using a TRAP staining kit (WAKO, Osaka, Japan). Osteoclasts with more than three nuclei were defined as mature osteoclasts, and the number of mature osteoclasts was analyzed using ImageJ software (v1.54i) [30].

TRAP staining of the femur sections of control and *ZC4H2* KO mice were carried out according to the instructions provided with the TRAP staining kit (WAKO, Osaka, Japan). The femoral sections were deparaffinized, rehydrated and stained with a freshly prepared TRAP staining solution at room temperature for 30 min. The sections were then counterstained with methyl green, mounted, and scanned using a digital slide scanning system (Olympus Corporation, Tokyo, Japan). The number of TRAP-positive osteoclasts was analyzed using ImageJ software [30].

### 2.10. F-Actin Ring Formation Assay

BMDM were induced to differentiate into osteoclasts for 7 days, followed by cell fixation, permeabilization, and blocking. The cells were then incubated overnight at 4 °C with Alexa Fluor™ 647-phalloidin (Thermo Fisher Scientific, Waltham, MA, USA). After incubation, the cells were washed 4 times with phosphate-buffered saline (PBS). Images were captured using an IX73 fluorescence microscope (Olympus Corporation, Tokyo, Japan), and the number of F-actin rings was quantified using ImageJ software.

### 2.11. Scanning Electron Microscopy

BMDM cells were seeded on bovine bone slices and induced to differentiate into osteoclasts for approximately one week. After removing the culture medium, pure water was added and left at room temperature for 10 min to swell and detach the cells from the bone surface. The bone slices were then gently wiped with absorbent cotton, followed by ultrasonic treatment for 4 min. The slices were dehydrated with a gradient series of alcohols to absolute ethanol and air-dried at room temperature. The samples were attached to the sample holder using conductive adhesive and thoroughly dried under vacuum. Gold sputtering was performed for approximately 45 s, and the samples were scanned and imaged using a scanning electron microscope (Sigma 500, Zeiss, Oberkochen, Germany) with an accelerating voltage of 10 kV.

### 2.12. ELISA Assay of CTX-1

The detection of CTX-1 content in mouse serum was performed using an ELISA kit (CUSABIO, Wuhan, China) according to the product instructions. The optical density (OD) was measured at 450 nm using a microplate reader. The results were analyzed using CurveExpert 1.4 (Hyams Development, Chattanooga, TN, USA).

### 2.13. Cell Counting Kit-8 (CCK-8) Assay

Cells were seeded at a density of 1000 cells/well in a 96-well plate, with 100 μL of complete medium added to each well. Then, 10 μL of CCK-8 was added to each well at 0, 2, 4, and 6 days, mixed, and incubated for 2 h. The absorbance at 450 nm was measured using a microplate reader. The OD values of blank wells (without cells, only medium and CCK-8) were used for calibration, followed by statistical analysis.

### 2.14. BrdU Incorporation Assay

Cells were seeded in a 24-well plate and cultured for 1–2 days. Then, BrdU (10 μM, Sigma, B5002) was added to the medium and incubated for 45 min. The medium was removed, and the cells were washed three times with PBS. Cells were then permeabilized with 0.3% Triton X-100, followed by the addition of 2N HCl and incubation at room temperature for 30 min. The cells were washed 4 times with PBS, then blocked with 10% goat serum for 1 h. Subsequently, cells were incubated with BrdU primary antibody (Sigma, B8434-25UL) and corresponding secondary antibody. After thorough washing, cells were stained with DAPI for nuclear staining, and finally observed and photographed using an IX73 fluorescence microscope (Olympus Corporation, Tokyo, Japan).

### 2.15. Statistical Analysis

All experiments were repeated at least three times, and the data were analyzed using GraphPad Prism 7.0 (GraphPad Software, San Diego, CA, USA) and presented as means ± standard deviation (SD). Unpaired two-tailed Student’s *t*-test or two-way analysis of variance (ANOVA) was used to determine the statistical significance. *p* < 0.05 was identified as statistically significant. n.s. represents no significant difference (*p* > 0.05).

## 3. Results

### 3.1. Loss of ZC4H2 Reduces Long Bone Calcification in Mice

**Figure 1:**
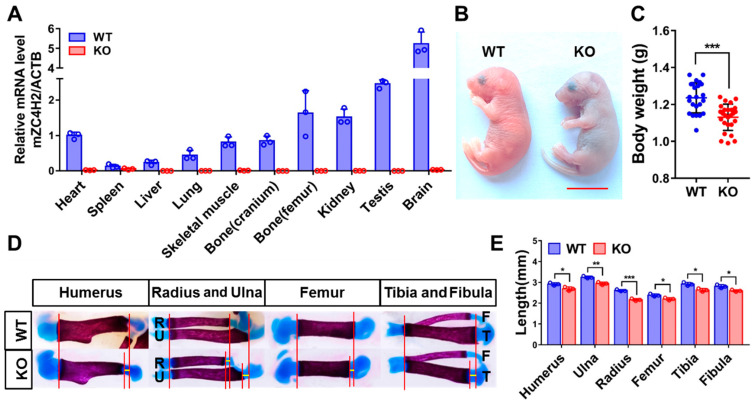
Knockout of *ZC4H2* affects bone development in mice. (**A**) The expression levels of *ZC4H2* in different tissues of P0 wild type and KO mice detected by qRT-PCR. (**B**) *ZC4H2* knockout mice are perinatal lethal and slightly smaller in size. (**C**) The body weight of P0 mice in the *ZC4H2* KO group was significantly reduced (WT: n = 23, KO: n = 26). (**D**) Alcian blue/Alizarin red staining of the long bones of neonatal control and *ZC4h2* KO mice. (**E**) The length of Alizarin red-stained regions between proximal and distal epiphyseal cartilages in each skeletal element was measured using NIS-Elements software (v5.10.00). Data represent mean ± SD, * *p* < 0.05, ** *p* < 0.01, *** *p* < 0.001. Scale bar, 1 cm in (**B**).

We first analyzed the expression of *ZC4H2* in different tissues of newborn mice (P0) using qRT-PCR. The results showed that *ZC4H2* is widely expressed in many tissues, strongest in the brain, and moderate in the skeletal muscle and bone (both cranium and femur, Figure 1A), which were lost in the *ZC4H2* knockout (KO) mice. As previously reported [11], the *ZC4H2* knockout mice were neonatal lethal (Figure 1B). We noticed that the neonatal *ZC4H2* embryos were slightly smaller, with lower body weight compared with wild-type littermates (Figure 1C). Histological analysis revealed no clear alterations in the morphology of the long bone of *ZC4H2*-deficient mice. We analyzed the calcification status of the long bones of the limbs in the newborn *ZC4H2* knockout and control embryos by Alcian Blue (which stains the cartilage) and Alizarin Red S (which stains the calcified bone) staining. Although the total length of the long bones (humerus, radius, ulna, femur, and tibia) of the *ZC4H2* knockout embryos were similar to those of the wild-type ones, the length of their calcified parts was generally shorter than those in the WT (Figure 1D,E).

### 3.2. Loss of ZC4H2 Reduces Femoral Bone Mass

**Figure 2:**
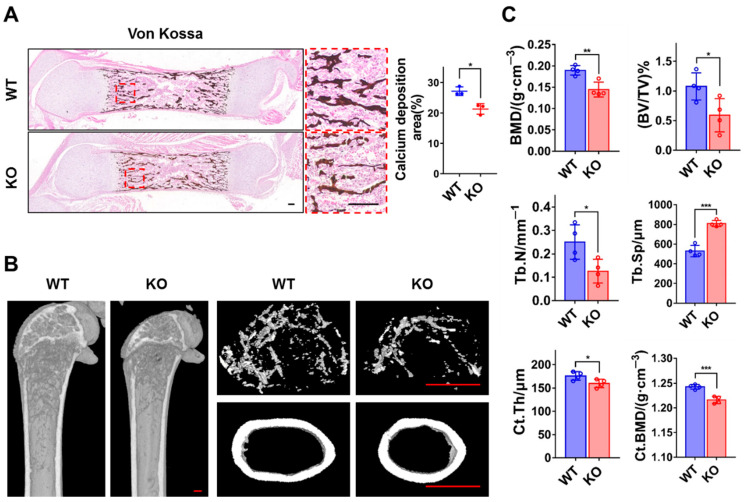
Loss of *ZC4H2* leads to reduced femoral bone mass in mice. (**A**) Von Kossa staining shows reduced calcium salt deposition in the femurs of P0 mice in the *ZC4H2* KO group. (**B**,**C**) Micro-CT analysis shows decreased bone density in 3-month-old mice in the *ZC4H2* TAM-KO group. In (**C**), the results are presented as bone mineral density (BMD), bone volume fraction (BV/TV), trabecular number (Tb.N), trabecular separation (Tb.Sp), cortical bone thickness (Ct.Th), and cortical bone mineral density (Ct.BMD). Data represent mean ± SD, * *p* < 0.05, ** *p* < 0.01, *** *p* < 0.001. Scale bar, 100 μm in (**A**); 200 μm in (**B**).

We further analyzed the effect of loss of *ZC4H2* on calcium deposition in the femur using von Kossa staining. The results revealed lower bone mass in the *ZC4H2* KO femur than in the control (Figure 2A). To study the potential effect of *ZC4H2* in postnatal bone development, we crossed the *ZC4H2*^fl/Y^ mice with a tamoxifen inducible Rosa26-CreERT2 line, and the *ZC4H2*^fl/Y^; Rosa26-CreERT2 mice were injected with tamoxifen at 1 week old to induce *ZC4H2* deletion. The bone mass of the femur of the TAM-*ZC4H2* KO and control mice were analyzed by micro-CT at 3 months old. Compared to the control group, the femoral bone in the KO group exhibited an osteoporosis-like phenotype, with reduced BMD, BV/TV, and Tb.N, increased Tb.Sp, and a slight reduction in Ct.Th and Ct.BMD (Figure 2B,C).

### 3.3. Loss of ZC4H2 Does Not Affect Osteoblast Differentiation 

The above data support that loss of *ZC4H2* leads to reduced bone mass, which could be due to inhibited osteogenesis or enhanced bone resorption. We therefore tested the effects of loss of *ZC4H2* on the differentiation of osteoblasts and osteoclasts using in vitro models. 

**Figure 3:**
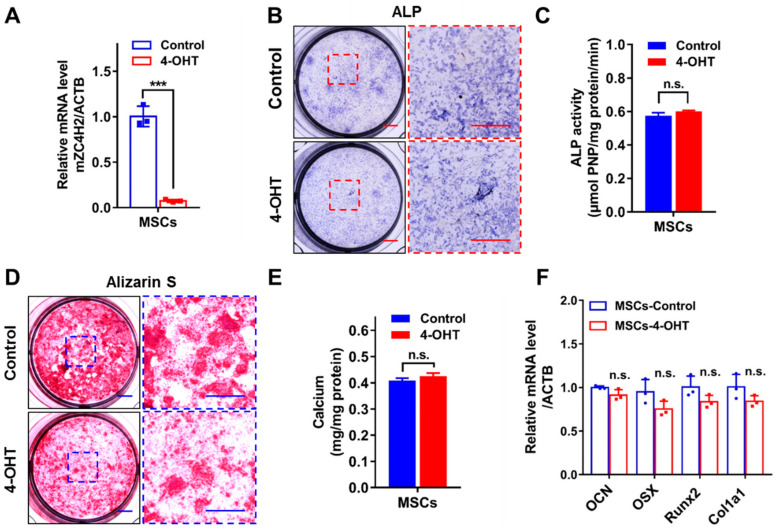
The effect of *ZC4H2* knockdown on osteogenic differentiation of MSCs. (**A**) 4-OH-tamoxifen (4-OHT) treatment efficiently induced loss of *ZC4H2* expression in the MSCs (*ZC4H2*^fl/Y^; Rosa26-CreERT2) as detected by qRT-PCR at 24-h. (**B**,**C**) ALP staining and quantitative analysis of osteogenic differentiation in control and *ZC4H2* KO MSCs. (**D**,**E**) ARS staining and quantitative analysis of osteogenic differentiation in control and *ZC4H2* KO MSCs. (**F**) The expression levels of genes related to osteogenic differentiation in differentiated control and *ZC4H2* KO MSCs. Data represent mean ± SD, *** *p* < 0.001. Scale bars, 2 mm in (**B**,**D**).

Bone marrow mesenchymal stem cells (MSCs) were isolated from *ZC4H2*^fl/Y^; Rosa26-CreERT2 mice at 6–8 weeks of age and induced into osteoblasts with or without tamoxifen treatment to induce deletion of *ZC4H2*. Addition of 4-OH-tamoxifen efficiently reduced the expression of *ZC4H2* in MSCs (Figure 3A). Alkaline phosphatase (ALP) and Alizarin red S (ARS) staining were conducted to analyze the osteogenic differentiation states. The results showed that loss of *ZC4H2* had no significant effect on the osteogenic differentiation of MSCs (Figure 3B–E). In the histochemical assays, the staining of ALP and ARS may seem more reduced in the KO group than in the control cells (Figure 3B,D). However, this is largely due to the reduced cell numbers because of the inhibitory effect of *ZC4H2* knockout on MSCs proliferation (see below). Indeed, in the quantified assays normalized to total protein levels (Figure 3C,E), no clear difference was observed between these two groups. We examined the expression of a group of osteogenic differentiation associated genes (*OCN*, *OSX*, *Runx2*, and *Col1a1*) by qRT-PCR. No significant change in their expression was observed in the *ZC4H2* KO group (Figure 3F). These results suggest that loss of *ZC4H2* does not affect osteogenesis in general.

### 3.4. Loss of ZC4H2 Promotes Osteoclastogenesis and Bone Resorption

**Figure 4:**
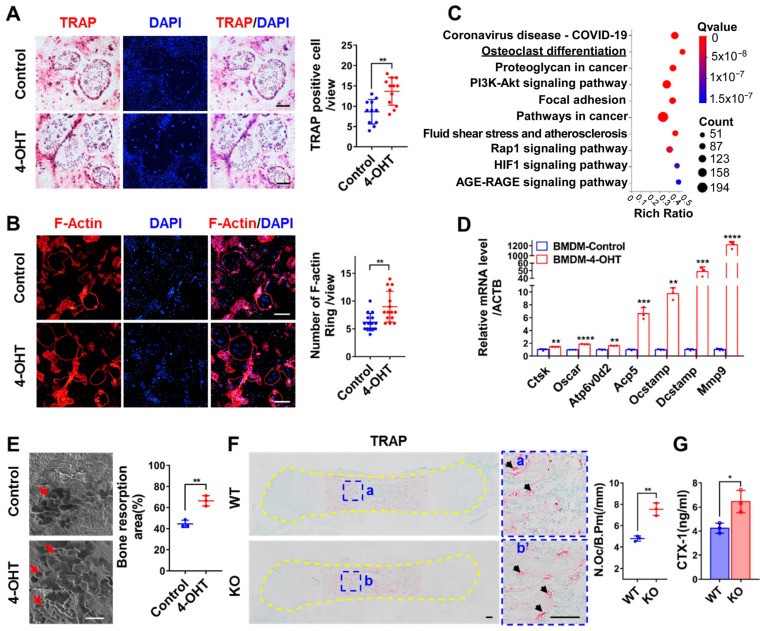
Loss of *ZC4H2* promotes osteoclast differentiation and bone resorption. (**A**) TRAP staining and quantitative analysis of multinuclear osteoclasts in control and *ZC4H2* KO (4-OHT) BMDM upon osteoclast differentiation. (**B**) F-actin staining and quantitative analysis of osteoclasts differentiation in control and *ZC4H2* KO BMDM. (**C**) RNA-Seq and KEGG pathway enrichment analysis of control and *ZC4H2* KO BMDM upon osteoclast differentiation. (**D**) qRT-PCR analysis of the expression of osteoclast differentiation associated genes in the differentiated control and *ZC4H2* KO BMDM. (**E**) Scanning electron microscopy results showing the areas of bone resorption pits (red arrowheads) on bovine bone slices induced by differentiated control and *ZC4H2* KO BMDM. (**F**) TRAP staining results showing the number of osteoclasts (arrowheads in a’, b’) in femur sections of P0 mice in control and KO groups. The number of osteoclasts per bone perimeter (N.Oc/B.Pm(/mm)) were measured with ImageJ software. (**G**) ELISA results showing the serum levels of CTX-1 in neonatal control and *ZC4H2* KO mice. Data represent mean ± SD, * *p* < 0.05, ** *p* < 0.01, *** *p* < 0.001, **** *p* < 0.0001. Scale bars, 200 μm in (**A**,**B**), 50 μm in (**E**), 100 μm in (**F**).

To examine the effects of *ZC4H2* knockout on osteoclast differentiation, BMDM were isolated from *ZC4H2*^fl/Y^; Rosa26-CreERT2 mice, and osteoclast differentiation assays were carried out in the presence or absence of 4-OH-tamoxifen. Addition of 4-OH-tamoxifen efficiently reduced the expression of *ZC4H2* in BMDM. Tartrate resistant acid phosphatase (TRAP) staining was used to visualize the multi-nucleated osteoclasts. The number of osteoclasts increased clearly in the *ZC4H2* KO group compared with the control cells (Figure 4A). This is further supported by the increase in the F-actin rings which represent active osteoclasts (Figure 4B). An RNA-Seq analysis was carried out on the control and *ZC4H2* KO BMDM upon osteoclast differentiation. Annotation of the differentially expressed genes showed clear enrichment in the osteoclast differentiation pathway as well as the PI3K-Akt signaling pathway (Figure 4C). In qRT-PCR analysis, the expression of the osteoclast differentiation associated genes (*Ctsk*, *Oscar*, *Atp6v0d2*, *Acp5*, *Ocstamp*, *Dcstamp*, and *Mmp9*) increased collectively, especially that of *Dcstamp* and *Mmp9* (Figure 4D). In bone resorption assays on bovine bone slices, the osteoclast group from *ZC4H2* KO BMDM produced more resorption pits than would be derived from control BMDM (Figure 4E), supporting increased bone resorption activity of the *ZC4H2* KO group. 

We tested whether this is also the case in vivo. TRAP staining of the P0 femur slice revealed more TRAP^+^ osteoclasts in the *ZC4H2* KO mice than in controls (Figure 4F). We also checked the serum levels of CTX-1 (a marker of osteoclastic activity) in P0 *ZC4H2* KO mice. The results showed clearly increased serum CTX-1 levels in the *ZC4H2* KO mice (Figure 4G), suggesting increased osteoclastic activity. These data support that loss of *ZC4H2* promotes osteoclastogenesis and bone resorption in vitro and in vivo.

### 3.5. Loss of ZC4H2 Inhibits Proliferation of MSCs and BMDM

**Figure 5:**
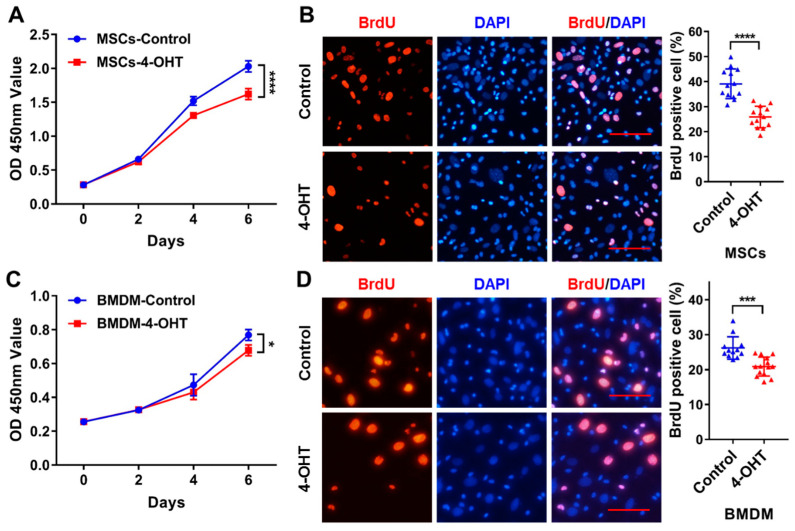
Knockdown of *ZC4H2* inhibits the proliferation activity of MSCs and BMDM. (**A**) Growth curves of control and *ZC4H2* KO MSCs assessed by CCK-8 proliferation assay. (**B**) BrdU incorporation assays and statistical results in control and *ZC4H2* KO MSCs. (**C**) Growth curves of control and *ZC4H2* KO BMDM assessed by CCK-8 proliferation assay. (**D**) BrdU incorporation assays and statistical results in control and *ZC4H2* KO BMDM. Data represent mean ± SD, * *p* < 0.05, *** *p* < 0.001, **** *p* < 0.0001. Scale bars, 200 μm in (**B**,**D**).

Using MSCs and BMDM derived from the *ZC4H2*^fl/Y^; Rosa26-CreERT2 mice, we tested the effects of 4-OH-tamoxifen induced deletion of *ZC4H2* on their proliferation using Cell Counting Kit-8 (CCK-8) and BrdU incorporation assays. CCK-8 assays showed significantly reduced cell proliferation of both the MSCs and BMDM on day 6 when *ZC4H2* was knocked down (Figure 5A,C), which was consistent with the lower BrdU incorporation rate in both cells (Figure 5B,D).

## 4. Discussion

In this report, we analyzed the potential roles of *ZC4H2* in bone development in mice. We showed that *ZC4H2* is expressed in different bone tissues, and loss of *ZC4H2* reduces bone calcification and leads to an osteoporosis-like phenotype. In an in vitro assay, knockdown *ZC4H2* had showed no clear effect on the osteogenic differentiation of mesenchymal stem cells, but the proliferation of the cells was inhibited. We cannot rule out the contribution of *ZC4H2* on osteogenesis in vivo through its effects on cell proliferation. Interestingly, we showed that *ZC4H2* knockout enhances osteoclast differentiation in mice. We utilized mouse bone marrow-derived macrophages to establish in vitro models for osteoclast differentiation. Morphological and transcriptomic analysis showed that *ZC4H2* knockdown significantly enhanced osteoclast differentiation and the expression of related genes, leading to increased bone resorption. We further confirmed that the number of osteoclasts in the long bone of *ZC4H2* knockout mice was increased, as well as the expression of the serum bone resorption/osteoporosis marker CTX-1. Thus, our work unveils a specific role of *ZC4H2* in regulating osteoclast differentiation and bone resorption activity, providing new clues for the pathology for ZARD. It would be of great interest to test whether bone resorption activities might be increased in ZARD patients, which could potentially contribute to the development of joint contracture, although this is not obvious in the *ZC4H2* KO mice. 

How *ZC4H2* is involved in osteoclast differentiation remains to be elucidated. The first question is whether *ZC4H2* regulates osteoclastogenesis through RNF220. Osteoclasts mainly originate from bone marrow monocyte–macrophage cell lines. We previously showed that RNF220 is expressed in bone marrow macrophages, which is responsive to pathogenic infection and IFN signaling. RNF220 mediated STAT1 ubiquitination contributed significantly to STAT1 activation and innate immune responses [31]. STAT1 signaling has been shown to be a negative regulator of osteoclast differentiation [32]. RNF220 is also involved in Shh, BMP, and Wnt signaling in different contexts [10,11,12], which are all involved in osteoclastogenesis. It is important to test whether osteoclast differentiation is also affected in RNF220 knockout mice. We cannot rule out the possibility that *ZC4H2* might work independently of RNF220 in osteoclastogenesis, which remains to be investigated in the future.

In summary, this study revealed that *ZC4H2* regulates bone resorption activity by inhibiting osteoclast differentiation in mice, providing new clues regarding the pathology of bone related phenotypes of ZARD.

## Figures and Tables

**Table 1:** Primer sequences used for qRT-PCR.

Gene	Forward Sequence	Reverse Sequence
*ACTB*	GGCTGTATTCCCCTCCATCG	CCAGTTGGTAACAATGCCATGT
*ZC4H2*	AAAGATCAAGGCCCGTTTG	TTGTATTCTTTCAGGTGCCTCTC
*OSX*	ACTCATCCCTATGGCTCGTG	GGTAGGGAGCTGGGTTAAGG
*OCN*	ACTCCGGCGCTACCTTGGAGCC	GCAGGGTTAAGCTCACACTG
*Runx2*	CCTAGTTAGAGTGGTAGCAGA	ACAGACAACGAAGAAAGTTCC
*Col1a1*	GCTCCTCTTAGGGGCCACT	CCACGTCTCACCATTGGGG
*Ctsk*	CTCGGCGTTTAATTTGGGAGA	TCGAGAGGGAGGTATTCTGAGT
*Mmp9*	CTGGACAGCCAGACACTAAAG	CTCGCGGCAAGTCTTCAGAG
*Acp5*	CACTCCCACCCTGAGATTTGT	CATCGTCTGCACGGTTCTG
*Dcstamp*	GGGGACTTATGTGTTTCCACG	ACAAAGCAACAGACTCCCAAAT
*Ocstamp*	CTGTAACGAACTACTGACCCAG	CCCAGGCTTAGGAAGACGAAG
*Atp6v0d2*	CAGAGCTGTACTTCAATGTGGA	AGGTCTCACACTGCACTAGGT
*Oscar*	CCTAGCCTCATACCCCCAG	CGTTGATCCCAGGAGTCACAA

## Data Availability

The data that support the findings of this study are available from the corresponding author upon reasonable request.
